# Toll-like receptors, chemokine receptors and death receptor ligands responses in SARS coronavirus infected human monocyte derived dendritic cells

**DOI:** 10.1186/1471-2172-10-35

**Published:** 2009-06-08

**Authors:** Helen KW Law, Chung Yan Cheung, Sin Fun Sia, Yuk On Chan, JS Malik Peiris, Yu Lung Lau

**Affiliations:** 1 Department of Paediatrics and Adolescent Medicine, Hong Kong Jockey Club Clinical Research Centre, Li Ka Shing Faculty of Medicine, The University of Hong Kong, Pokfulam, Hong Kong, PR China; 2 Department of Microbiology, Hong Kong Jockey Club Clinical Research Centre, Li Ka Shing Faculty of Medicine, The University of Hong Kong, Pokfulam, Hong Kong, PR China

## Abstract

**Background:**

The SARS outbreak in 2003 provides a unique opportunity for the study of human responses to a novel virus. We have previously reported that dendritic cells (DCs) might be involved in the immune escape mechanisms for SARS-CoV. In this study, we focussed on the gene expression of toll-like receptors (TLRs), chemokine receptors (CCRs) and death receptor ligands in SARS-CoV infected DCs. We also compared adult and cord blood (CB) DCs to find a possible explanation for the age-dependent severity of SARS.

**Results:**

Our results demonstrates that SARS-CoV did not modulate TLR-1 to TLR-10 gene expression but significantly induced the expression of CCR-1, CCR-3, and CCR-5. There was also strong induction of TNF-related apoptosis-inducing ligand (TRAIL), but not Fas ligand gene expression in SARS-CoV infected DCs. Interestingly, the expressions of most genes studied were higher in CB DCs than adult DCs.

**Conclusion:**

The upregulation of chemokines and CCRs may facilitate DC migration from the infection site to the lymph nodes, whereas the increase of TRAIL may induce lymphocyte apoptosis. These findings may explain the increased lung infiltrations and lymphoid depletion in SARS patients. Further explorations of the biological significance of these findings are warranted.

## Background

The SARS outbreak in 2003 provides a unique opportunity for the study of human response to a novel virus. The etiological agent was identified to be a coronavirus (CoV) originating from an animal reservoir [1,2]. Clinically, patients presented with an atypical pneumonia followed by diffuse alveolar damage, with mortality up to 52% in patients over 65 years old. Interestingly, the disease presentations of SARS were less severe in children than adults, and none of the infected children (< 12 years old) died of SARS [3,4]. Evidences in the literature, including ours, support the role of immune evasion in the severity and immunopathology of SARS (Reviewed by [5-7]), and we further suggested that the developmental status of the host immune system may be responsible for the age-dependence of disease severity in SARS.

In our previous report [8], we have demonstrated that dendritic cells (DCs), the key antigen presenting cells with crucial role in anti-viral immune responses, might also be involved in the immune escape mechanisms for SARS-CoV. There was entry and incomplete replication of SARS-CoV in DCs but there was no production of infectious virus released into the culture medium [8]. SARS-CoV infection did not lead to DCs apoptosis or DC maturation.

Interestingly, the SARS-CoV infected DCs showed low expression of antiviral cytokines (IFN-α, IFN-β, IFN-γ and IL-12p40), moderate upregulation of proinflammatory cytokines (TNF-α and IL-6) but significant upregulation of inflammatory chemokines (macrophage inflammatory protein (MIP)-1α/CCL3, regulated upon activation, normal T cell expressed and secreted (RANTES)/CCL-5, interferon-inducible protein of 10 kD (IP-10)/CXCL10 and monocyte chemotactic protein (MCP)-1/CCL2. We postulated that this lack of antiviral cytokine response against a background of intense chemokine upregulation could represent a mechanism of immune evasion by SARS-CoV.

In the current study, we focused on the receptor responses in SARS-CoV infected DCs and compared adult and cord blood (CB) DCs to establish possible explanation for the age dependent severity of SARS. DCs express a wide range of receptors for the recognition of conserved pathogen patterns as well as the induction of subsequent immune responses. Some Toll like receptors (TLRs) are expressed on the cell surface (TLR-1, TLR-2, TLR-4, TLR-5, TLR-6, TLR-10) while some are located within intracellular compartments (TLR-3, TLR-7, TLR-8, TLR-9) [9]. They are differentially expressed in different DC subsets and are modulated in response to a variety of stimuli [10,11]. Viral proteins may bind to TLR-2 or TLR-4, single stranded RNA binds to TLR-7 and TLR-8, and double stranded RNA binds TLR-3 while viral DNA binds to TLR-9. The binding of ligands to TLRs may trigger downstream signaling pathways that are involved in both the cytokine release during the primary induction of inflammation and secondary activation of anti-inflammatory mechanisms [12]. Cross talks between TLRs are common and the formation of TLR heterodimers allows a higher level of complexity in ligand-receptor binding and subsequent signaling.

The migration of DCs from peripheral tissues to lymph nodes is essential for antigen presentation and triggering of adaptive immune responses. The trafficking of DCs is regulated by chemokines in their microenvironment and their expression of chemokine receptors (CCRs). Differential expressions of CCRs are observed during DC maturation [13,14] and some viruses, such as herpes simplex virus (HSV), can block CCR expressions on DCs to alter their migratory properties [15]. There are redundancies in the chemokines and CCRs interactions as many different ligands bind the same receptor and many receptors bind the same ligand. For example, RANTES binds to CCR-1, CCR-3 and CCR-5, while MIP-1α also binds to CCR-1 and CCR-5 [16]. We determined if the expression of these receptors on DCs are altered by SARS-CoV and contribute to autocrine regulation of DC migration.

Death receptors (DRs) and their ligands also play important roles in innate and adaptive immune responses by regulating cell death and survival [17]. Well-characterized death receptor ligands (DRLs) include tumor necrosis factor (TNF)-α, FasL and TNF-related apoptosis-inducing ligand (TRAIL/Apo2L). Recently, several viruses, including measles virus [18], human immunodeficiency virus (HIV) [19], cytomegalovirus (CMV) [20], and herpes simplex virus (HSV) [21], were shown to induce TRAIL expression on DCs. These "killer DCs" may be involved in the killing of virus-infected cells or bystander lymphocytes and natural killer cells. In view of the lymphopenia observed in SARS patient, we determine if DRLs expression on DCs is modulated by SARS-CoV.

This study provides evidence that SARS-CoV does not alter the TLRs, but modulates CCRs and DLRs expression in DCs; and further suggests possible mechanisms of immune escape and amplification of immunopathology in SARS.

## Results

### SARS-CoV did not stimulate the gene expression of TLRs in immature DCs

**Figure 1:**
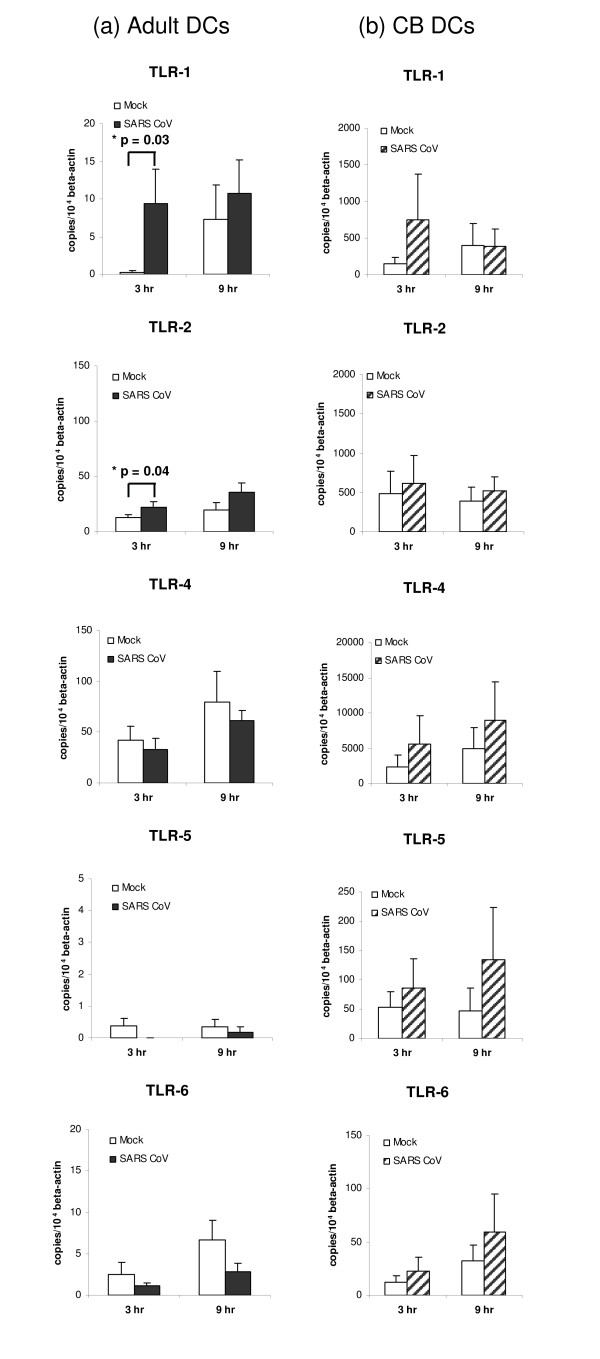
**Extracellular Toll-like receptors gene expression in SARS-CoV infected human immature DCs by quantitative RT-PCR**. Toll-like receptor (TLR)-1, TLR-2, TLR-4, TLR-5, TLR-6 and TLR-10 primarily expressed on cell surface. Their mRNA concentrations in adult (a) and CB (b) immature DCs were assayed at 3 h and 9 h after infection with SARS-CoV (MOI = 1). Mock infected cells were included as negative control. The concentrations were normalised to those of β-actin mRNA in the corresponding sample. In SARS-CoV infected adult DCs, significant upregulation of TLR-1 and TLR-2 at 3 h after infection was detected. The basal levels of extracellular TLRs gene expressions in CB DCs were high and no significant upregulation was detected after infection with SARS-CoV. Data are shown as mean ± SEM (adult n = 7; CB n = 5). The expression of TLR-10 was <1 copy per 10^4 ^β-actin genes (Data not shown).

Gene expression of extracellular TLRs (TLR-1, TLR-2, TLR-4, TLR-5, TLR-6) in DCs are summarised in Fig. 1. There was a low (<50 copies/per 10^4 ^β-actin), but significant upregulation of TLR-1 and TLR-2 in SARS-CoV infected adult DCs at 3 h post infection. A similar trend was observed in CB DCs but the difference did not reach statistical significance. In CB DCs, the basal gene expressions of TLR-1, TLR-2, and TLR-5 and the SARS-CoV induced TLR-2 and TLR-4 expression were significantly higher than that in adult DCs (Table 1). No expression of TLR-10 was detected in either adult or CB DCs (data not shown).

**Table 1:** CB immature DCs expressed significantly higher level of some receptor genes than adult immature DCs**

	**3 h p.i.**		**9 h p.i.**	
	**MOCK**	**SARS-CoV**	**MOCK**	**SARS-CoV**

TLR-1	p = 0.02	-	-	-

TLR-2	p = 0.003	p = 0.003	p = 0.003	p = 0.003

TLR-3	-	-	-	-

TLR-4	-	p = 0.03	-	-

TLR-5	p = 0.004	-	p = 0.04	-

TLR-6	-	-	-	-

TLR-7	p = 0.04	-	-	-

TLR-8	-	-	-	-

TLR-9	-	-	-	-

CCR-1	-	p = 0.02	-	p = 0.005

CCR-3	-	-	-	-

CCR-5	-	-	-	p = 0.02

CCR-7	p = 0.02	p = 0.01	-	p = 0.03

FasL	-	-	-	-

TRAIL	-	p = 0.005	-	p = 0.01

**Adult DCs (n = 7)

CB DCs (n = 5)

TLR, toll-like receptor; CCR, chemokine receptor; FasL, Fas ligand; TRAIL, Tumor necrosis factor (TNF)-related apoptosis-inducing ligand

**Figure 2:**
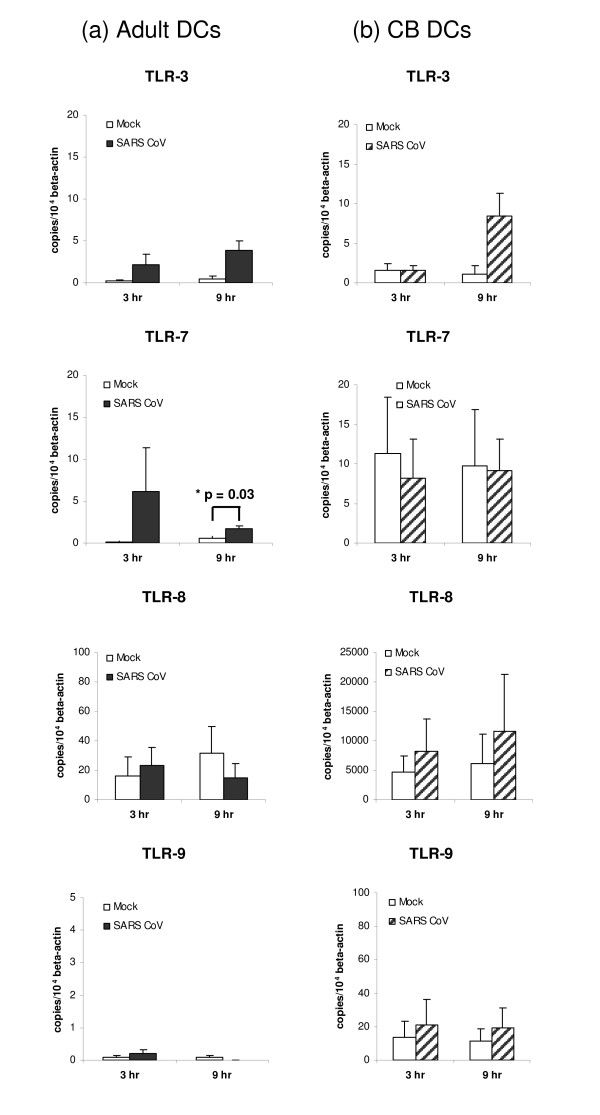
**Intracellular Toll-like receptors gene expression in SARS-CoV infected human immature DCs by quantitative RT-PCR**. Toll-like receptor (TLR)-3, TLR-7, TLR-8 and TLR-9 primarily expressed intracellularly. Their mRNA concentrations in adult (a) and CB (b) immature DCs were assayed at 3 h and 9 h after infection with SARS-CoV (MOI = 1). Mock infected cells were included as negative control. The concentrations were normalised to those of β-actin mRNA in the corresponding sample. In SARS-CoV infected adult DCs, low but significant upregulation of TLR-7 was detected at 9 h after infection. The basal levels of intracellular TLR-8 gene expression in CB DCs was very high and no significant upregulation was detected after infection with SARS-CoV. Data are shown as mean ± SEM (adult n = 7; CB n = 5).

Gene expression of intracellular TLRs (TLR-3, TLR-7, TLR-8, TLR-9) in DCs are summarised in Fig. 2. In adult DCs, the expression was low (average in range of 0 – 20 copies per 10^4 ^β-actin). The expression of TLR-3, TLR-7 and TLR-9 were also low in CB DCs (average in range of 0 – 20 copies per 10^4 ^β-actin). The basal gene expression of TLR-8 was high in CB DCs and the expression was nearly doubled after SARS-CoV infection at both 3 h and 9 h post infection. However, due to sample variations the difference between mock and SARS-CoV infected CB DCs was not statistically significant.

### SARS-CoV stimulated moderate expression of CCR genes in immature DCs

**Figure 3:**
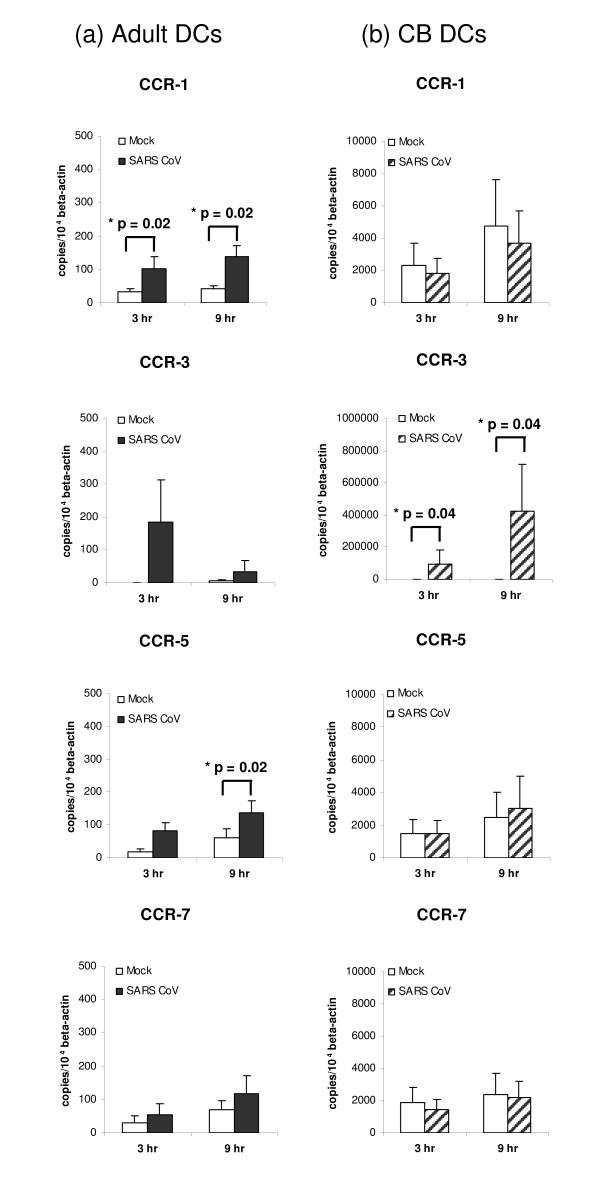
**Chemokine receptors gene expression in SARS-CoV infected human immature DCs by quantitative RT-PCR**. Chemokine receptors, CCR-1, CCR-3, CCR-5, CCR-7, mRNA concentrations in adult (a) and CB (b) immature DCs were assayed at 3 h and 9 h after infection with SARS-CoV (MOI = 1). Mock infected cells were included as negative control. The concentrations were normalised to those of β-actin mRNA in the corresponding sample. In SARS-CoV infected adult DCs, significant upregulations of CCR-1, CCR-3 and CCR-5 were detected. In the CBDCs, only upregulation of CCR-3 was detected. Data are shown as mean ± SEM (adult n = 7; CB n = 5).

Comparing the mock and SARS-CoV infected adult DCs, there was moderate induction of CCR-1, CCR-3, CCR-5 and CCR-7 at both 3 h and 9 h post infection (Fig. 3) but only that observed for CCR-1 and CCR-5 reached statistical significance. It is important to note that at 3 h post infection, the CCR-3 expression in mock infected adult DCs was very low (average <1 copies per 10^4 ^β-actin) but it increased to 183.91 ± 128.35 copies per 10^4 ^β-actin after SARS-CoV infection. In SARS-CoV infected CB DCs, a strong and significant upregulation was observed for CCR-3 at both 3 h and 9 h post infection. In general, the expressions of these CCRs in CB DCs were significantly higher than in adult DCs (Table 1).

### SARS-CoV upregulated TRAIL but not FasL gene expression in immature DCs

**Figure 4:**
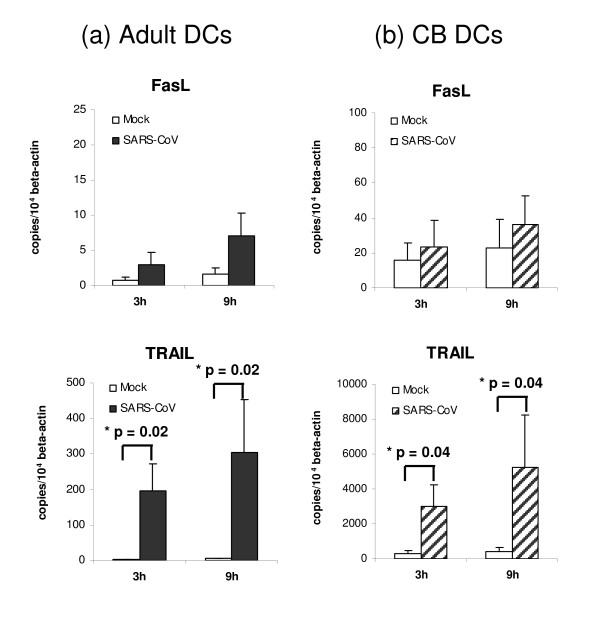
**Death Receptor Ligand gene expression in SARS-CoV infected human immature DCs by quantitative RT-PCR**. Death receptor ligands, FasL and TRAIL, mRNA concentrations in adult (a) and CB (b) immature DCs were assayed at 3 h and 9 h after infection with SARS-CoV (MOI = 1). Mock infected cells were included as negative control. The concentrations were normalised to those of β-actin mRNA in the corresponding sample. In both SARS-CoV infected adult DCs and CB DCs, significant upregulation of FasL mRNA was not detected. However, there was significant upregulation of TRAIL gene expression. Data are shown as mean ± SEM (adult n = 7; CB n = 5).

We also assayed the expression of DRLs in SARS-CoV infected DCs as they may have an impact on the killing of bystanding immune cells. The gene expressions of FasL and TRAIL in DCs were summarised in Fig. 4. In both adult and CB DCs, FasL gene expression was low (~0–40 copies per 10^4 ^β-actin) and there was a slight and insignificant induction by SARS-CoV. In the contrary, a strong induction of TRAIL gene expression was observed in SARS-CoV infected DCs at both 3 h and 9 h post infection. The upregulated TRAIL expression in CB DCs were significantly higher than that in adult DCs (Table 1).

## Discussion

Dendritic cells are professional antigen presenting cells that play an important role as sentinels at the infection site by interacting directly with pathogens. Some pathogens hijack different parts of the immune pathways involving DCs to enhance their survival. In the present study, we demonstrate that SARS-CoV does not have significant effect on the expression of TLRs on adult or CB DCs (Figs. 1 &2). However, there was significant upregulation of CCR-1 and CCR5 in SARS-CoV infected adult DCs and CCR-3 in SARS-CoV infected CB DCs which may be related to the autocrine regulation of DC migration (Fig. 3). Moreover, we demonstrate a significant upregulation of TRAIL on both adult and CB DCs (Fig. 4). We hypothesize that the upregulation of chemokine receptors may facilitate the autocrine mobilisation of DCs away from the infection sites and the upregulation of death receptor liganads may result in apoptosis of other immune cells, leading to immunosuppression and lymphopenia.

TLRs are expressed on many cell types and their expression on different DC subsets have been characterised. Visintin et al. [22] measured TLR expression on immature human DCs by monoclonal antibodies binding, but the expression was very low, corresponding to a few hundred molecules or less in immature DCs. Subsequent studies of TLRs expressions were based on comparing RNA expression quantitatively and some reports for immature DCs have been controversial. Our detection of TLR-1 to TLR-9 expressions in mock infected adult DCs were consistent with that reported by Jarrossay et al. [10] and the low level of TLR-10 was similar to that reported by Kadowaki et al. [11].

Viruses may subvert the TLR response to evade the host defense by: (a) producting specific viral particles that block TLR function, (b) blocking TLR recognition and (c) stimulating viral replication through TLRs [23]. Viral infections, by RSV or influenza A, have been shown to upregulate TLR-3 and TLR-4 expression in host airway epithelial cells, leading to increased signalling activity and proinflammatory cytokine production [23]. More recently, TLR-4 was shown to be upregulated in mice with acid-induced or inactivated avian influenza-induced lung injuries; and TLR-4 deficient mice showed less severe acute lung injuries to these challenges [24]. We, therefore, determined if SARS-CoV may modulate the TLRs expression on DCs. In this study, there was no induction nor suppression of TLRs in SARS-CoV infected DCs (Figs. 1 &2). The variations between samples were high, but the expression levels of some extracellular TLRs (TLR-1, TLR-2, TLR-4, TLR-5) and some intracellular TLRs (TLR-7, TLR-8) in CB DCs were higher than that in adult DCs (Table 1). Further investigation is needed to determine if the expression is responsible for the slightly higher Type I inferferon expression reported in CB DCs [8] and to identify any correlation with less severe SARS in children. Interestingly, impaired immune responses to TLR-3 and TLR-4 ligands have been reported in CB DCs [25].

The majority of TLR research is focussed on the downstream signalling pathways triggered by the binding of specific ligands to TLRs. Activation of TLRs induce signalling cascades which activate transcription factors and gene expression for anti-inflammatory responses [9]. The identification of such ligands may have therapeutic applications for the treatment of SARS. In a recent study on the control of coronavirus infection by plasmocytoid DCs (pDCs), it was demonstrated that mouse hepatitis virus (MHV) induces Type I IFN response and the recognition of MHV is mediated by TLR7 [26]. The authors demonstrated that SARS-CoV also induced a strong Type I interferon response in human pDCs and proposed that TLR7 may play a similar role in SARS-CoV infection. Another group developed a recombinant mouse-adapted SARS-CoV (rMA15) that was lethal in BALB/c mice and demonstrated that MyD88 played an important role in SARS-CoV pathogenesis [27]. Both groups suggested the involvement of TLR7/MyD88/IFNα dependent signaling and further exploration is needed to determine if TLR7 agonist may elicit potent antiviral effects against SARS-CoV infection.

To date, approximately 50 chemokines and 20 CCRs have been identified in humans [16] and they play important roles in inflammation and infectious diseases [28]. In our previous study, there was significant increase in the inflammatory chemokines (MIP 1α, RANTES, IP-10 and MCP-1) in SARS-infected DCs [8]. In addition, we have shown that SARS patients who have inherited the high-production gene allele of RANTES have more deaths [29]. In this study, we determined whether the expressions of representative CCRs (CCR1, CCR-3, CCR-5 and CCR-7) are also upregulated by SARS. Indeed, there was significant induction of CCR-1, CCR-3 and CCR-5 mRNA expression in SARS-CoV infected DCs (Fig. 3) suggesting the possibility of a autocrine loop in facilitating the trafficking of DCs. Therefore, further investigation into therapeutic strategies which aim at reducing chemokine and CCRs expresssion are warranted.

Among the CCRs studied, the upregulation of CCR-3 is the strongest (Fig. 3). It has been reported that the expression of CCR-3, unlike CCR-5 and CCR-7, are independent of the maturation status of DCs [13,14]. Further investigation is needed to determine which mechanism is involved in CCR-3 upregulation. In addition to cell trafficking, CCR-3 has also been reported to function as a death receptor on B cells [30]. Whether CCR-3 expression is related to the characteristic antibody responses in SARS and the alternative function of CCRs on SARS-CoV infected DCs will need to be explored further.

CCR-7 has been identified as a key molecule for the establishment of central and peripheral tolerance. It is strongly upregulated in herpesvirus infected T cells [31], EBV infected B cells [32] and mature DCs [14]. In addition, microarray analysis of PBMC infected with SARS-CoV have shown an upregulation of CCR7 [33]. In this study, we only detected a slight but insignificant change in CCR-7 expression in adult SARS-CoV infected DCs (Fig. 3). This observation is consistent with previous findings that SARS-CoV did not stimulate DC maturation [8,34]. However, this is in contrary to observations made by others [35,36]. We speculate as these immature DCs migrate to the lymph nodes, they may act as regulatory DCs which are less effective for the activation of allogeneic CD4+ T cells than normal DCs [37] or they may drive the development of regulatory T cells [38]. These SARS-CoV-driven regulatory immature DCs may facilitate the immune evasion of SARS-CoV [39].

Interestingly, we detected significantly higher levels of chemokines and CCRs genes in CB DCs than in adults DCs. Based on the function of chemokines on cell trafficking, more severe infiltration of cells to the lungs would be expected in children. On the contrary, SARS was less severe in children than adults [3,4]. The age-dependency of disease severity in SARS merits further studies to elucidate the underlying mechanisms. The development of animal models for SARS has been difficult because relevant signs of clinical illness and death cannot be reproducible in most animal models [40]. Recently, aged BALB/c mice have shown to develop more severe disease after SARS infection [41] and have lower vaccine efficacy than young mice [42]. The comparison of SARS-CoV infected senescent mice with adult mice can be extended to younger mice and knock out models for the study of age-dependency in the pathogenesis of SARS. A recent study has shown that mice deficient in either CCR1, CCR2 or CCR5 exhibited more prominent airway epithelial cell apoptosis and more severe lung pathology. This suggests that CCRs may be playing different roles at the site of infection and in the trafficking of immune cells [27].

Using real time quantitative assay, we have shown significant upregulation of TRAIL gene expression in avian influenza (H5N1) infected macrophages [43]. Similarly, SARS-CoV infected DC also showed a "killer DC' phenotype with high expression of TRAIL (Fig. 4). Comparing the two studies, the level of TRAIL expression in SARS-CoV infected adult DCs (~300 copies/10^4 ^β actin genes) was lower than that in H5N1 infected adult macrophages (~1800 copies/10^4 ^β actin genes). TRAIL expression in SARS-CoV infected CB DCs was the highest (~5000 copies/10^4 ^β actin genes). Further investigation is needed to confirm the cytotoxic function of SARS-CoV infected DCs on immune cells. Based on the possible DC migration induced by chemokines and the role of TRAIL in inducing lymphocyte apoptosis, our findings may explain the white pulp atrophy in the spleen and lymphoid depletion in lymph nodes of SARS patients. The mechanism involved in children may be more complicated and it is important to determine if the CB DCs are functionally immature in spite of the high expression of chemokines, CCRs and TRAIL. More recently, TRAIL expression is also reported to be upregulated after the binding of HIV to TLR-7 [44] or activation of TLR-8 [45]. Hence the high TRAIL expression in SARS-infected CB DCs may also be related to their TLR-8 expression.

## Conclusion

This study has characterized important receptor responses of DCs to SARS-CoV. The suggested mechanisms need to be substantiated by further research involving a larger sample size for different pathways as pathogens may have multiple ways to modify the physiology of DCs to their advantage.

## Methods

### Samples

Adult blood samples were from the white cell fraction of blood donated to the Hong Kong Red Cross by normal healthy volunteers. Human umbilical cord blood (CB) samples were collected from the placenta of normal full-term uncomplicated pregnancies. Informed consent was obtained from the mothers prior to delivery. The protocol was approved by the Institutional Review Board of the University of Hong Kong/Hospital Authority Hong Kong West Cluster [EC1473-00].

### Cell separation

Blood mononuclear cells were isolated from whole blood by centrifugation, using Ficoll-Hypaque gradients (Pharmacia Biotech, Uppsala, Sweden), washed, and labeled with immunomagnetic antibodies. Positive selection was performed according to manufacturer's specification (Miltenyi Biotec, Bergisch Gladbach, Germany) as in previous experiments [8]. Isolated CD14+ monocytes from the positive fraction were resuspended in RPMI 1640, supplemented with 50 IU/ml penicillin and 50 μg/ml streptomycin and 10% fetal bovine serum (Invitrogen, Grand Island, USA). Cell viability, as measured by trypan blue exclusion, was more than 95%. The purity of the isolated cells as measured by flow cytometry was consistantly between 90% to 95%.

### Generation of DCs in vitro

CD14+ monocytes were cultured in the presence of IL-4 (10 ng/ml; R&D, Minneapolis, USA) and GM-CSF (50 ng/ml; R&D, Minneapolis, USA) for 7 days at 37°C in a humidified atmosphere containing 5% CO_2 _as in our previous study [8]. The cultures were fed with fresh medium and cytokines on Day 3 and cell differentiation was monitored by light microscopy. On Day 5, DCs were harvested, centrifuged, washed and adjusted to 1 × 10^6 ^cells/mL before virus infection.

### Virus preparation, titration and infection

Laboratory procedures involving live viruses was performed in biosafety level-3 containment. SARS-CoV, strain HKU-39849 [1] was cultured in fetal rhesus kidney-4 (FRhK-4) cells. The cell culture supernatant was harvested, centrifuged to remove cell fragments, aliquoted and kept frozen at -70°C. SARS-CoV titre of the stock virus was determined by infection of FRhK-4 cells. Cytopathic changes on FRhK-4 cells was monitored every day up to 4 days and virus titre expressed as tissue culture infective dose (TCID_50_).

Cells were inoculated by SARS-CoV at a multiplicity of infection (MOI) of 1. The virus was allowed to be adsorbed for 1 hour at 37°C and unbound virus was washed off by excess volume of PBS (time = 0 h post infection). Mock infected cells were treated in parallel, except that virus was not added.

### Quantification of RNA expression by real-time quantitative PCR

Total RNA was extracted from ~1.5 × 10^5 ^cells harvested at 3 h and 9 h post infection by TRIzol Reagent (Invitrogen Life Technologies, USA). In later experiments, QiaShredder columns (Qiagen, Hilden, Germany) were used to ensure adequate homogenisation and RNA was extracted by the RNeasy Mini Kit (Qiagen, Hilden, Germany). Reverse transcription was performed on the DNase-treated total RNA using oligo (dT) primers and Superscript II reverse transcriptase (Invitrogen Life Technologies, USA) according to the manufacturer's recommendation. The cDNA synthesised were diluted (1:50) and quantified by real-time PCR using Taqman Technology (Applied Biosystems, CA, USA). Specific primers (Table 2) were used and non-specific reactions and primer-dimer artifacts have been minimised (as evaluated by gel electrophoresis). Detection of PCR product was based on Taqman fluorescence signal. Standard curves were generated using serial dilutions of plasmids (~10 – 10^10 ^copies) containing cloned sequences involved. Results were calculated as the number of targeted molecules/μL cDNA. To standardise results for variability in RNA and cDNA quantity and quality, we express the results as the number of target copies per 10^4 ^copies of β actin gene, which was determined previously [8].

**Table 2:** PCR primers and probes

Genes	Sequences	Sizes (bp)
TLR-1	(F) 5' CAG TGT CTG GTA CAC GCA TGG T 3'	105
	(P) 5' (FAM) CAC ATG CTT TGC CAT CCA AAA TTA G (TARMA) 3'	
	(R) 5' TTT CAA AAA CCG TGT CTG TTA AGA GA 3'	

TLR-2	(F) 5' TGT GAA GAG TGA GTG GTG CAA GT 3'	78
	(P) 5' (FAM) TGA ACT GGA CTT CTC CCA TTT CCG TCT TT (TARMA) 3'	
	(R) 5' ATG GCA GCA TCA TTG TTC TCA T 3'	

TLR-3	(F) 5' CCT GGT TTG TTA ATT GGA TTA ACG A 3'	82
	(P) 5' (FAM) CAT ACC AAC ATC CCT GAG CT (MGB) 3'	
	(R) 5' TGA GGT GGA GTG TTG CAA AGG 3'	

TLR-4	(F) 5' CAC TCG ATG TCA TTC CAA AGT TAT TG 3'	88
	(P) 5' (FAM) TAC TAA GTA ATG ACT GTC ATG AAA GCA GCA T (TARMA) 3'	
	(R) 5' AGA GTG CCC CCT TTA AAC AAA TT 3'	

TLR-5	(F) 5' TGC CTT GAA GCC TTC AGT TAT G 3'	77
	(P) 5' (FAM) CCA GGG CAG GTG CTT ATC TGA CCT TAA CA (TARMA) 3'	
	(R) 5' CCA ACC ACC ACC ATG ATG AG 3'	

TLR-6	(F) 5' GAA GAA GAA CAA CCC TTT AGG ATA GC 3'	88
	(P) 5' (FAM) TGC AAC ATC ATG ACC AAA GAC AAA GAA CCT (TARMA) 3'	
	(R) 5' AGG CAA ACA AAA TGG AAG CTT 3'	

TLR-7	(F) 5' TTA CCT GGA TGG AAA CCA GCT AC 3'	72
	(P) 5' (FAM) AGA TAC CGC AGG GCC TCC CGC (TARMA) 3'	
	(R) 5' TCA AGG CTG AGA AGC TGT AAG CTA G 3'	

TLR-8	(F) 5' AGC GGA TCT GTA AGA GCT CCA TC 3'	107
	(P) 5' (FAM) CCT GAC AAC CCG AAG GCA GAA GGC (TARMA) 3'	
	(R) 5' CCG TGA ATC ATT TTC AGT CAA GAC 3'	

TLR-9	(F) 5' GCA GTC AAT GGC TCC CAG TTC 3'	116
	(P) 5' (FAM) CCC ACA ATA AGC T (MGB) 3'	
	(R) 5' GCG GTA GCT CCG TGA ATG AGT G 3'	

TLR-10	(F) 5' TTA TGA CAG CAG AGG GTG ATG C 3'	152
	(P) 5' (FAM) TTG ACC CCA GCC ACA ACG ACA CTG (TARMA) 3'	
	(R) 5' CTG GAG TTG AAA AAG GAG GTT ATA GG 3'	

CCR-1	(F) 5' CCC AAT GGG AAT TCA CTC ACC A 3'	200
	(P) 5' (FAM) CCT GCA GCC TTC ACT TTC CTC ACG AAA (TARMA) 3'	
	(R) 5' CAA ACG GAC AGC TTT GGA TT 3'	

CCR-3	(F) 5' CAC AGC AGA GCC GGA ACT C 3'	75
	(P) 5' (FAM) TGT GTT TTA GGT CAG ATG CAG AAA AT (TARMA) 3'	
	(R) 5' CTC CTT GGT CCT TCC TCT TTA GG 3'	

CCR-5	(F) 5' ACT GCA AAA GGC TGA AGA GC 3'	204
	(P) 5' (FAM) ACT GAC ATC TAC CTG CTC AAC CTG GCC A (TARMA) 3'	
	(R) 5' CGA TTG TCA GGA GGA TGA TG 3'	

CCR-7	(F) 5' ACG GAC GAT TAC ATC GGA GA 3'	195
	(P) 5' (FAM) ACA CGA CCA GCC CAT TGC CCA GT (TARMA) 3'	
	(R) 5' GGT CAT GGT CTT GAG CCT CT 3'	

FasL	(F) 5' ACC TCA AGG GGG ACT GTC TT 3'	170
	(P) 5' (FAM) ACA TGG TTG TGA CCT GAG GAT TTA AGG GAT GG (TARMA) 3'	
	(R) 5' TTA GTT TCA CCG ATG GCT CA 3'	

TRAIL	(F) 5' CCC CTG CTG GCA AGT CAA 3'	68
	(P) 5' (FAM) TGG CAA CTC CGT CAG CTC GTT (TARMA) 3'	
	(R) 5' CCT CAG AGG TTC TCA AAA TCA TCT T 3'	

F, forward primers; R, reverse primers; P, Taqman probes

TLR, toll-like receptor; CCR, chemokine receptor; FasL, Fas ligand; TRAIL, Tumor necrosis factor (TNF)-related apoptosis-inducing ligand

### Statistical analysis

All data were expressed as mean ± SEM. All samples were paired and differences between groups were analyzed by paired Student t test or the non-parametric equivalents using the Instat software (GraphPad Software, Inc. San Diego, CA, USA).

## Abbreviations

SARS: severe acute respiratory syndrome; CoV: coronavirus; DC: dendritic cell; CB: cord blood; FRhK-4 cells: fetal rhesus kidney 4 cells; MOI: multiplicity of infection; TLR: toll-like receptor; CCR: chemokine receptor; DRL: death receptor ligand

## Authors' contributions

HKWL prepared the dendritic cells. SFS and YOC performed the experiments involving the virus in the Biosafety Level 3 laboratories. HKWL and SFS carried out the molecular studies. HKWL, CYC, JSMP and YLL participated in the design of the study and analyzed data. HKWL and YLL drafted the manuscript. All authors read and approved the final manuscript.
